# Autologous Bone Marrow Mononuclear Cell Therapy for Autism: An Open Label Proof of Concept Study

**DOI:** 10.1155/2013/623875

**Published:** 2013-08-25

**Authors:** Alok Sharma, Nandini Gokulchandran, Hemangi Sane, Anjana Nagrajan, Amruta Paranjape, Pooja Kulkarni, Akshata Shetty, Priti Mishra, Mrudula Kali, Hema Biju, Prerna Badhe

**Affiliations:** ^1^Department of Medical Services and Clinical Research, NeuroGen Brain and Spine Institute, Surana Sethia Hospital and Research Centre, Sion Trombay Road, Chembur, Mumbai 400071, India; ^2^Department of Research & Development, NeuroGen Brain and Spine Institute, Surana Sethia Hospital and Research Centre, Sion Trombay Road, Chembur, Mumbai 400071, India; ^3^Department of NeuroRehabilitation, NeuroGen Brain and Spine Institute, Surana Sethia Hospital and Research Centre, Sion-Trombay Road, Chembur, Mumbai 400071, India

## Abstract

Cellular therapy is an emerging therapeutic modality with a great potential for the treatment of autism. Recent findings show that the major underlying pathogenetic mechanisms of autism are hypoperfusion and immune alterations in the brain. So conceptually, cellular therapy which facilitates counteractive processes of improving perfusion by angiogenesis and balancing inflammation by immune regulation would exhibit beneficial clinical effects in patients with autism. This is an open label proof of concept study of autologous bone marrow mononuclear cells (BMMNCs) intrathecal transplantation in 32 patients with autism followed by multidisciplinary therapies. All patients were followed up for 26 months (mean 12.7). Outcome measures used were ISAA, CGI, and FIM/Wee-FIM scales. Positron Emission Tomography-Computed Tomography (PET-CT) scan recorded objective changes. Out of 32 patients, a total of 29 (91%) patients improved on total ISAA scores and 20 patients (62%) showed decreased severity on CGI-I. The difference between pre- and postscores was statistically significant (*P* < 0.001) on Wilcoxon matched-pairs signed rank test. On CGI-II 96% of patients showed global improvement. The efficacy was measured on CGI-III efficacy index. Few adverse events including seizures in three patients were controlled with medications. The encouraging results of this leading clinical study provide future directions for application of cellular therapy in autism.

## 1. Introduction

Autism spectrum disorders (ASD) are a group of heterogeneous neurodevelopmental disorders characterized by deficits in verbal and nonverbal communication, social interaction, and presence of stereotypical repetitive behavior. The genetic, environmental, and immunological factors have been attributed as underlying causes, though its exact etiology is unknown. The incidence of autism has increased to a great extent, which may be due to increased awareness leading to an early and accurate diagnosis or due to perinatal complications, genetic factors, environmental factors, and lifestyle changes. Presently, the worldwide incidence is 12 per 1000 children [1]. Despite its increasing rate, currently autism remains untreatable. The available options of behavioral therapy and pharmacological and supportive nutritional treatments are only palliative. Medical therapy is directed towards the neuropsychiatric disorders associated with ASDs. Commonly prescribed medicines are selective serotonin reuptake inhibitors, antipsychotics, mood stabilizers, and psychostimulants. Methylphenidate may be used to treat attention deficit or hyperactivity. Anticonvulsants are used for seizures with autism [2]. However, the use of medications is limited by their side effects. There is a desperate need of a medical intervention to tackle the basic pathogenetic mechanisms. Several genes have been found to be associated with ASD. This provides the basis for treatment with gene therapy in future. Currently, for safe human gene therapy to be applied to this population, several areas need further research [3].

The major neurophysiological alterations are immune abnormalities and neural hypoperfusion, and its correlation with symptomatology has been reported [4]. Cellular therapy exerts potent angiogenetic and immunoregulatory effects along with other paracrine effects [5, 6]. Recently, cell transplantation has been shown to be safe and efficacious in several neurological disorders [7–10]. A variety of cellular therapies with different cell types and routes of administration is being explored. The major types are embryonic, umbilical cord, induced pluripotent, and adult stem cells. The use of adult stem cells is devoid of any ethical issues and can be obtained from bone marrow, adipose tissue, skin, dental pulp, and other sources. Bone marrow stem cells have been extensively studied and can be easily procured [8, 11]. The intrathecal route is an easy, safe, and direct approach to provide the cells to the brain without causing any neural tissue damage [12, 13]. 

Conceptually, cellular therapy should give beneficial clinical effects in patients with autism. The concept is based on its potential to counterbalance the core pathogenetic mechanisms of autism [4]. The aim of this study is to assess the safety, efficacy, and clinical effects of autologous bone marrow mononuclear Cells (BMMNCs) transplantation in patients with autism.

## 2. Materials and Methods

### 2.1. Study Design

This is an open label proof of concept study on the use of autologous BMMNCs transplantation in 32 patients with autism. The intervention included cellular therapy with intrathecal transplantation of autologous bone marrow derived mononuclear cells followed by occupational therapy, speech therapy, and psychological intervention. The primary objective was to document any adverse events and establish the safety of the intervention within a period of 26 months (December 2010 to February 2013). The secondary objective of the study was to evaluate the effects of the intervention on symptoms, disease severity, extent of disability, and functional impairment caused by autism.

### 2.2. Participants Eligibility Criteria and Recruitment

Patient selection was based on World Medical Association Helsinki Declaration for Ethical Principles for medical research involving human subjects [14]. A written informed consent was obtained from the parents of all patients. All the patients included in the study had confirmed diagnosis of autism according to the DSM-IV TR diagnostic criteria for autistic disorder. The exclusion criteria were presence of acute infections such as HIV/HBV/HCV, malignancies, bleeding tendencies, renal failure, severe liver dysfunction and other acute medical conditions such as respiratory infection and pyrexia.

### 2.3. Intervention

#### 2.3.1. Preintervention Assessment

The Institutional Committee for Stem Cell Research and Therapy (IC-SCRT) granted the ethical approval for the treatment protocol. An informed consent was taken from the parents of all the patients. Prior to intervention, all the patients underwent a thorough clinical examination with serological, biochemical, and hematological tests. Magnetic Resonance Imaging (MRI) of the brain and Electroencephalography (EEG) were also conducted in all patients. In view of the clinical improvements observed, a preintervention PET-CT (Positron Emission Tomography-Computed Tomography) scan was introduced at a later stage of the study.

#### 2.3.2. Procurement of Autologous BMMNCs

Patients were administered Granulocyte Colony Stimulating Factor (GCSF) 48 hours and 24 hours before the harvest and transplantation of BMMNC [15]. On the day of the transplantation, bone marrow was aspirated under general anesthesia in the operation theatre with aseptic precautions. Approximately, 100 mL of bone marrow (varying between 80 mL and 100 mL, based on the age and body weight) was aspirated from the region of anterior superior iliac spine using the bone marrow aspiration needle and collected in the heparinized tubes.

#### 2.3.3. Isolation of BMMNCs

The aspirate was then transferred to the laboratory where the mononuclear cells were separated by the density gradient method. CD34+ counting was done by Fluorescence activated cell sorting (FACS) [16]. The MNCs were checked for viability (average viability count was found to be 97%).

#### 2.3.4. Mode of Cell Transplantation

The separated autologous BMMNCs were immediately injected on the same day, intrathecally using an 18 G Tuohy needle and epidural catheter at the level between fourth and fifth lumbar vertebrae. The average numbers of cells injected were 8.19 × 10^7^. Simultaneously 20 mg/kg body weight methyl prednisolone in 500 mL Ringer Lactate was given intravenously to enhance survival of the injected cells. Patient was monitored for any adverse events.

#### 2.3.5. Post-BMMNCs Transplantation Therapy

All the patients underwent extensive therapy under the guidance of experts. This includes occupational therapy interventions based on sensory integrative approach, activities of daily living (ADL) training, psychological intervention based on behavior modification techniques, speech therapy, and specific dietary recommendations. The therapy protocol was planned out specifically for individual patients, as per the detailed assessment done before the therapy.

The therapy sessions conducted during the stay in the hospital were recorded and compiled in a CD-ROM which was given to all the patients at discharge. A therapy plan to be followed at home was designed for all the patients. Patients were advised to continue therapy at home under the supervision of a professional. Follow-up assessment was done at regular intervals over a period of 26 months.

### 2.4. Outcome Measures

To assess the safety of the intervention, outcome measures were used to monitor any major or minor adverse events through the entire duration of followup. Patients were counseled regarding the probable adverse events during informed consent. Recording of adverse events during the hospital stay was done by a health professional whereas after discharge it was recorded, as reported by the parents or primary care givers.

#### 2.4.1. Monitoring Procedure Related Adverse Events

Acute procedural adverse events, associated with cell aspiration and injection via lumbar puncture, were stringently monitored over 5–7 days after intervention. Body temperature, blood pressure, respiratory rate, and heart rate were recorded at regular intervals. Patients were examined thoroughly for signs of spinal headache, motor or sensory loss, incontinent bowel and bladder, damage to brain or spinal cord, respiratory distress, cardiac failure, and allergic reaction. Aspiration and injection sites were examined every day for pain, bleeding, and signs of infection. Signs and symptoms of any anesthesia complications, back pain associated with lumbar puncture, headache, nausea, and vomiting were checked regularly. All the minor acute procedural adverse events were treated medically prior to the discharge of the patients from the hospital. Patients were examined thoroughly at each followup for neurological deficits exhibiting clinically as motor or sensory loss that was not present before intervention. A detailed history was also taken to rule out any transient neurological symptoms.

#### 2.4.2. Monitoring Cellular Transplantation Related Adverse Events

During the stay in the hospital, signs and symptoms of any allergic reaction were monitored at regular intervals. Long term major and minor adverse events were monitored to establish the safety of stem cell transplantation. A detailed history was also taken to rule out presence of any seizures. If any seizure was reported, EEG was done for evaluation and managed by a neurologist. Any clinical deterioration of the core symptoms of autism was recorded by Indian Scale for Assessment of Autism (ISAA), Clinical Global Impression (CGI) scale, Functional Independence Measure (FIM), and Wee-FIM scales (see Supplementary Material available online at doi: http://dx.doi.org/10.1155/2013/623875 (Appendices I, II, III, and  IV)).

#### 2.4.3. Monitoring the Effects After Intervention

Outcome measures used for the effects of intervention were CGI, ISAA, FIM, and Wee-FIM scales. CGI scale was used for measuring the change in the severity of the disease, overall improvement, and the efficacy of the treatment. The efficacy component of CGI takes into consideration the improvements and side effects after the cellular transplantation. CGI-I scale for severity of illness was scored before cellular therapy and at follow-up visit. CGI-II scale for global improvement and CGI-III scale for efficacy index were each measured at the time of follow-up assessment. The scores recorded by experienced clinicians during the latest visit were used for the analysis. The CGI-I scale is an ordinal scale and reflects a clinical comparison of patients' severity of symptoms with the typical clinical presentation. Although CGI scale is grossly perceived as a subjective scale the psychometric properties of the CGI scale have been calculated earlier for various psychiatric disorders. The scale shows good reliability, validity, and sensitivity for these disorders [17–19]. The CGI scale for autism has been previously used successfully as an outcome measure in various trials [20–22].

The effect of cell transplantation on the extent of disability was measured using the ISAA. Most of the available diagnostic tools and outcome measures have been designed for the western population. In a disorder like autism where communication and social behavior is impaired, an assessment tool that takes into account the social and cultural context is crucial. Although derived from CARS, ISAA has been designed for Indian population. It is divided into six domains. There are forty questions which are comprehensive and specific to the difficulties experienced by children with autism. It grades the symptoms in ascending order of intensity of symptoms on an ordinal scale of 1 to 5. The content, construct and concurrent validity, internal consistency and test-retest reliability, and sensitivity and specificity of ISAA were studied by the members of the expert committee for the development of assessment tool for autism. ISAA was thus found to be a valid tool, with good reliability and high sensitivity and specificity [23]. ISAA scores were marked during the assessment before the stem cell transplantation and at every follow-up assessment after transplantation, however the scores marked at the latest assessment were considered for comparison and analysis.

To measure the functional independence in activities of daily living, Wee-FIM for children below the age of 8 years and FIM for above 8 years were used. FIM has been shown to be a valid and reliable tool [24]. In addition to these outcome measures various symptoms were monitored through structured interviews and assessment by clinicians.

In view of clinical improvements, PET-CT scanning was introduced to observe functional neuroimaging changes in brain. Measurements were taken before and six months after the transplantation. PET studies were performed using the Siemens Biograph mCT with 64-slice high speed scanner, 3D PET True V wide detector (Siemens-CTI, Knoxville, Tenn., USA), which has an intrinsic resolution of 0.6 mm full width at half maximum (FWHM) and the images of 45–50 contiguous transverse planes with a field of view of 21.6 cm axial PET FOV with True V.

Standard conditions were maintained during all of the [^18^F1] FDG PET scans. Time duration between injection of the dye and scanning was constant at 30 minutes for all the patients and at all instances. The scan room was dimly lit and there was minimal auditory stimulation during injection and scanning period. PET scan was performed with patients lying in supine position with eyes closed to reduce any activity related confounding effect. Imaging data were processed using proprietary Scenium Software before final image reconstruction.

### 2.5. Statistical Analysis

#### 2.5.1. Description of Sample

The demographic data for all the patients was recorded and analyzed. Mean age in years at the time of intervention, mean age in years at the time of diagnosis, and mean time duration in months at which the patients were followed up were calculated. Median CGI and FIM scores were calculated. Median score was calculated for the total ISAA score and the individual domains.

#### 2.5.2. Statistical Tests

The pre- and postintervention scores of CGI-I, total ISAA scores, scores of ISAA domains, Wee-FIM, and FIM were compared using Wilcoxon signed rank test for matched pairs with the predetermined level of significance at 0.05. Percentage analysis was conducted for the CGI-II and CGI-III scales and for individual symptoms as described in ISAA scale. Statistical analysis was carried out using SPSS 17.0 software.

## 3. Results

### 3.1. Description of the Sample

There were total 32 patients with 24 (75%) males and 8 (25%) females. The age of the population ranged from 3 to 33 years with a mean age of 10.5 (5.6) years. They were diagnosed on an average at 7.1 (5.2) years before the intervention. The follow-up period ranged between 5 and 26 months with a mean followup of 12.7 (7.1) months. The baseline ISAA scores ranged from 148 to 160 with a median of 115.5 (18.33) (due to the even number of patients), CGI-I scores ranged from 3 to 6 with a median of 4.5 (1), FIM scores ranged from 48 to 118 with a median of 77 (18), and Wee-FIM scores ranged from 18 to 118 with a median of 76 (24) (Table 1).

### 3.2. Statistical Analysis

There was a statistically significant difference between the pre- and post-CGI-I scores (*P* = 0.001) and total ISAA scores (*P* < 0.001) (Table 2). There was no statistically significant difference between the FIM scores (*Z* = −1.841, *P* = 0.066) and Wee FIM scores (*z* = −1.000, *P* = 0.317). All the ISAA domains showed a statistically significant (*P* < 0.05) reduction after intervention (Table 3).

### 3.3. Percentage Analysis

**Figure 1:**
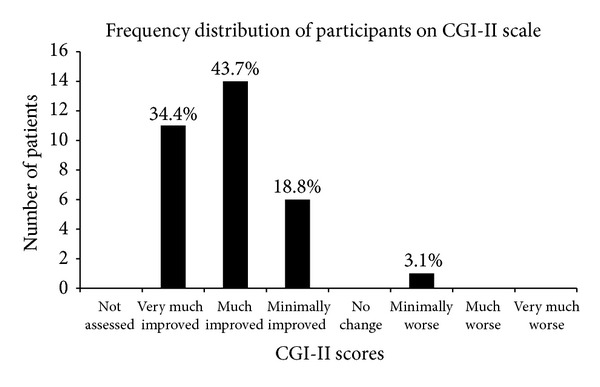
Frequency distribution of participants on CGI-II scale. This figure shows the percentage of people showing varying degrees of improvement after cellular transplantation.

**Figure 2:**
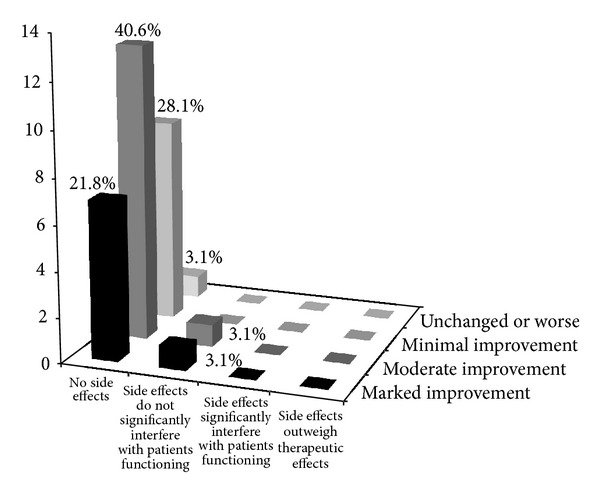
Frequency distribution of the participants on the CGI-III scale. This figure demonstrates the percentage of people on varying degrees of efficacy of the intervention. This takes into consideration the side effects and the benefits observed.

Overall the ISAA score reduced in 29 out of 32 (90.6%) patients. On CGI-II scale 96.9% showed global improvement. Out of these 43.7% patients showed much improvement and 34.4% patients showed very much improvement. There were 18.8% with minimal improvement and 3.1% patients with minimal worsening (Figure 1). According to the CGI-III scale 93.8% patients had no side effects and only 6.2% patients had minimal side effects that did not interfere with function. 21.8% patients showed marked improvement with no side effects, 40.6% patients showed moderate improvement with no side effects, 28.1% patients showed minimal improvement with no side effects, 3.1% patients showed marked improvement with side effects that did not interfere with patients functioning, and 3.1% patients showed moderate improvement with side effects that did not interfere with patients functioning (Figure 2).

Here we describe the number of patients showing improvement in a particular symptom as indicated by the percentage in the parenthesis.

In the domain of social relationships and reciprocity, 29 out of 32 (90.6%) patients showed improvement. Patients showed improved eye contact (70%), social smile (56%), and reaching out to others (42%). They were able to take turns in social interaction (55%), respond to social or environmental cues (46%), and maintain peer relationships (55%). There was a decrease in three symptoms: inability to relate to people (30%), tendency to remain aloof (59%), and engagement in solitary and repetitive play activities (41%).

Improved emotional responsiveness was observed in 18 out of 32 (56%) patients. Inappropriate emotional responses (42%), exaggerated emotions (48%), engaging in self-stimulating emotions (55%), and getting excited or agitated for no apparent reason (56%) decreased. Lack of fear of danger (37%) did not reduce significantly; yet, few patients were reported to have shown some decrease.

Under the speech, language, and communication domain there was an improvement observed in 25 patients out of 32 (78%). A significant reduction was seen in echolalic speech (54%), engaging in stereotyped repetitive use of language (53%), production of infantile squeals or unusual noises (52%), inability to initiate or sustain conversation with others (45%), and inability to grasp the pragmatics of the conversation (43%) and speech regression (50%). It is noteworthy that few patients also showed clinical improvement in symptoms of difficulty in using nonverbal language or gestures (32%), using of jargon or meaningless words (28%), using of pronoun reversal (27%).

Behavior patterns of 21 out of 32 patients (66%) improved. Hyperactivity or restlessness (71%) and engaging in stereotype and repetitive motor mannerisms (65%) decreased significantly. Attachment to inanimate objects (50%), throwing temper tantrum (42%), aggressive behavior (48%), self-injurious behavior (53%), and insisting on sameness (43%) also reduced. 

Sensory aspects improved in 14 out of 32 patients (44%). Unusual sensitivity to sensory stimuli (50%), staring into space for long periods of time (25%), difficulty in tracking objects (46%), unusual vision (62%), insensitivity to pain (26%), and responses to objects or people unusually by smiling or touching or tasting (39%) reduced significantly.

Cognitively they showed improved consistency in attention and concentration and response time. 71% patients showed better attention and concentration, 45% patients showed reduction in the delay in responding.

Functional neuroimaging in the form of PET-CT scan in eight patients noted the following changes. After cellular therapy changes in the glucose metabolism in the form of FDG uptake were observed in the frontal and parietal lobes of six patients, occipital and temporal lobes, of five patients and cerebellum of four patients. Further analysis of other regions showed changes in medial temporal lobe of four patients; amygdala, hippocampus, and parahippocampus of three patients; and cingulate, paracingulate area, and basal ganglia of five patients.

### 3.4. Adverse Events Monitoring

#### 3.4.1. Procedure Related Adverse Events

During the procedure there were no complications in the operation theatre. None of the patients had signs and symptoms of local or systemic infection, meningismus, any neurological deficit, parasthesias in the lower limb, any nerve root damage, or hematoma at the site of aspiration and allergic reaction. During the hospital stay, few patients showed procedure related minor adverse events like spinal headache (3.6%), nausea (10.7%), vomiting (17.9%), backache and pain at the site of injection (7.1%), and aspiration (7.1%). These adverse events were controlled with medication and resolved within one week. There were no procedure related major adverse events (Table 4).

#### 3.4.2. Cellular Transplantation Related Adverse Events

Adverse events related to cellular transplantation were transient minimal increase in hyperactivity (6 out of 32), persistent increase in hyperactivity (1 out of 32), and seizures (3 out of 32). Six patients had transient increase in hyperactivity at three-month followup. One patient showed persistent increase in hyperactivity at six months that did not interfere with the global clinical improvement. One patient with marked symptomatic improvement and one patient with moderate symptomatic improvement developed seizures after therapy, which were controlled with antiepileptic medications. One out of four patients who had a previous history of seizures showed increased episodes of seizures for a few weeks which were controlled with increased dosages of his antiepileptic medications (Table 5). Despite this the patient showed moderate improvements with no symptomatic deterioration. Patients who had normal EEG before cellular transplantation did not have seizures after cellular transplantation.

## 4. Discussion

An important aim of regenerative medicine is to find a definitive treatment for incurable disorders. In this effort, various conditions such as Parkinson's disease, spinal cord injuries, muscular dystrophy, myocardial infarction, and stroke are studied and have shown beneficial therapeutic effects [7–9]. Recently, cell-based therapy for autism has been explored by researchers to a great extent [4–6]. 

Autism is one of the most complicated neurodevelopmental disorders with a very high prevalence rate [1]. Its exact etiology and pathophysiology remains poorly understood. The numerous biochemical abnormalities detected in autism are oxidative stress, endoplasmic reticulum stress, mitochondrial dysfunction, decreased methylation, underproduction of glutathione, intestinal dysbiosis, and toxic metal burden [25]. The environmental factors like organophosphates and heavy metals are also attributed to the origin of the disease [26]. Genetics involve multiple mutations in various genes resulting in its varied phenotype. These mutations may result in structural or molecular or functional defects in synaptogenesis [27].

A range of findings have suggested autism as a disorder of growth of the neural systems and connections, likely to be responsible for the underdevelopment of functions such as communication, behavior, and socialization [28]. U. Frith and C. Frith (2010) proposed a social brain hypothesis to explain theory of mind deficits in ASD [29]. The social brain concept tries to localize the complex social perception to superior temporal sulcus (STS), amygdala, orbital frontal cortex (OFC), and fusiform gyrus (FFG) [30]. The key roles implicated are STS region in analysis of perception, FFG in face detection and recognition, OFC in social reinforcement and reward processes, and the amygdala in analysis and regulation of emotions [31]. These areas form neural interconnections to establish a pathway from perception to action [32]. Neuroimaging studies have indicated dysfunction in the social brain areas and aberrant neuronal circuitry in autism [33, 34].

Recently, brain hypoperfusion and immune dysfunction have been recognized as two major pathogenetic mechanisms associated with autism. Hypoperfusion results in hypoxia, abnormal metabolite or neurotransmitter accumulation leading to neural tissue damage. The degree of hypoperfusion is proportional to the severity of the symptoms of autism. The extent of hypoxia was shown to be inversely correlated to Intelligent Quotient (IQ) [35]. Immune dysfunction is an imbalance in pro-inflammatory and anti-inflammatory factors. The raised macrophage product neopterin, TNF-alpha, MCP-1, and IFN-gamma indicate an augmented inflammatory response. In addition, deficient levels of anti-inflammatory cytokines such as IL-10 and TGF-beta suggest lack of natural inhibitory feedback processes. Autoimmune mechanism is also thought to be causative due to detection of autoantibodies to myelin basic protein, Purkinje cells and gliadin extracted peptides, neurotrophic factors, and neuron-axon filament and glial fibrillary acidic protein [36, 37]. T cell and B cell abnormalities have been demonstrated with systemic T cell lymphopenia, decreased cell proliferation, and abnormal production of cytokines [38]. During the period of neurodevelopment, the deregulated immune activity may result in the neurological dysfunctions in autism [39, 40]. Ichim et al. (2007), in their review, have proposed the administration of stem cells as a novel treatment to address the core pathologies of autism [4].

Cellular therapy has the therapeutic potential to repair the damaged neural tissue at molecular, structural, and functional levels. The stem cells possess unique properties of self-renewal, transdifferentiation [41], and paracrine effects [15]. The paracrine action changes the local micromilieu by secretion of trophic factors like ciliary neurotrophic factor (CNTF), vascular endothelial growth factor (VEGF), and fibroblast growth factor (FGF). This stimulates the local repair by enhancing proliferation, cell recruitment, and maturation of endogenous stem or progenitor cells [42]. The CD34+ stem cells have the capacity to produce angiogenic factors and differentiate into endothelial cells themselves [43]. Therapeutic angiogenesis improves perfusion and clearance of toxic metabolites and reduces hypoxia.

Another important effect is immunomodulation through inhibition of T lymphocyte pro-inflammatory cytokine production (IL-1*β*, TNF-*α*, and INF-*γ*) and upregulation of anti-inflammatory IL-10 and TGF-beta. This counterbalances the aberrant immune systems and reduces neural damage with restoration of functions [44, 45].

### 4.1. Source Selection and Route of Administration

Several sources of stem cells have been identified such as fetal or embryonic, bone marrow, umbilical cord, adipose tissue and dental pulp [46]. Bone marrow derived stem cells are easily procured by a standard procedure [47] and its potency and safety has been well established without any ethical issues [8, 9]. The bone marrow comprises of a heterogeneous population of stem cells, encompassing hematopoietic stem cells (HSCs), mesenchymal stem cells (MSCs), endothelial progenitor cells (EPCs), and very small embryonic-like stem cells (VSELs) [48]. This offers advantage of variety effects of different cell types.

The choice of intrathecal route was guided by efficient delivery of cells to brain with a relatively less invasive and safe procedure. The injected cells are transported by CSF to the affected areas in the brain [49, 50]. Various mechanisms have been believed to lead to altered permeability of blood brain barrier allowing the transplanted cells to reach brain areas with marked inflammation [51, 52]. The intrathecal route enhances the possibility of maximal number of transplanted cells “homing” onto damaged sites.

### 4.2. Clinical Findings in This Study

Conceptually the cellular therapy mechanisms, as described above, address the core pathogenesis of autism. The novel findings on the molecular, cellular, neuroimmunological, and environmental factors contributing to the pathogenesis of autism provide a rationale for cellular therapy as a unique and potent tool. Therefore, as a proof of concept, we studied 32 cases of autism, which were treated with BMMNCs, intrathecally.

The study sample included a total of 32 participants of which 24 were males and 8 were females, which is a ratio of 3 : 1. The gender ratio is synonymous to findings in previous studies [53]. The study included children as well as adults with autism. A majority of the patients were undergoing rehabilitation therapy since the time of diagnosis. Severity of autism in the participants ranged from mild to severe, as measured on the ISAA.

The clinical results, stated above as evidence to the concept, are discussed here. With a good number of participants showing clinically significant improvements on the ISAA and its subcomponents, the patterns of symptomatic improvement were analyzed. We gather here a theoretical pattern of the improvements noted after cellular therapy. Immediately after the intervention, within one week, initiation or consistency of eye contact and minimal decrease in hyperactivity were observed. Restlessness, rocking, hand flapping, and jumping, which are motor behavior seen in relation to the sensory issues, were seen to reduce early after intervention and continue to do so even later. This aids in better participation during sessions for behavior management and speech therapy. Only a few patients showed an increase in the levels of hyperactivity which could be attributed to the challenges posed by a new social and physical environment at the hospital and the changes in their routine. Arousal and activity levels continued to normalize progressively over the next three to six months. The immediate effect of the decrease in hyperactivity is seen on the levels of attention and concentration. Attention span increases and patients begin to sit at one place for activities, attend to commands, and follow them. These enhance the quality and duration of therapy sessions attended and aids in learning new concepts. With the ability to follow commands emerging and the improved attention span, facilitation of meaningful communication becomes more effective. They later start communicating their needs and using nonverbal gestures too. Besides the sensory issues, behavior has also been attributed to their inability to communicate or express. It was observed that, as they start communicating and indicating their needs and with the appropriate reinforcement strategies, their abnormal behavior slowly fades away. They also began to initiate social interaction and engage in play, forming peer relationships.

**Figure 3:**
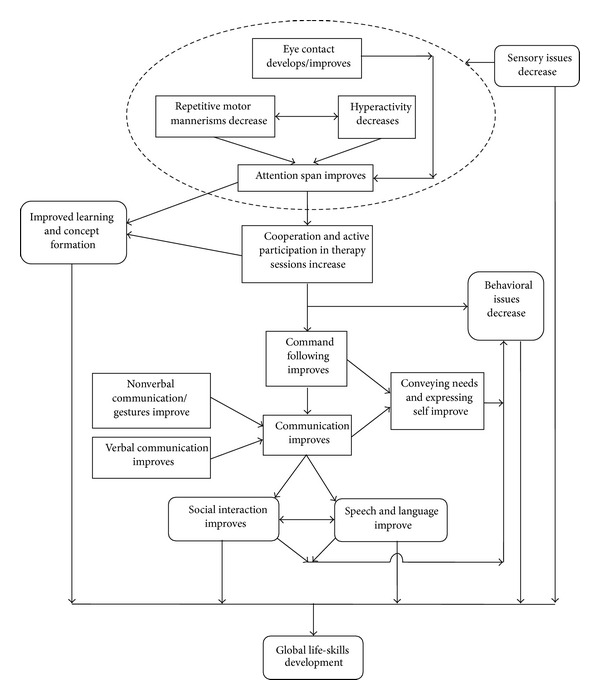
Schematic representation of clinical improvements after cellular therapy. This figure shows proposed theoretical outline of observed changes after cellular therapy.

Speech was found to have improved at later stages. Speech is a complex function requiring good listening skills, attention, auditory processing, comprehension, and motor co-ordination. All of these are affected in many individuals with autism. We believe that it requires longer duration of consistent therapy for significant improvements. Developmentally speech develops as monosyllable, bisyllables, words, phrases and lastly sentences. We have observed that in the patients treated with autologous BMMNCs followed by supportive therapies that individuals who were absolutely nonverbal (or mute) before therapy developed nonverbal communication. Individuals who had monosyllables progressed to bisyllables and so on (Figure 3).

Hence, significant improvements were noted in all the interrelated domains of the ISAA, namely, social relationships and reciprocity, emotional responsiveness, speech, language, and communication, behavior patterns, sensory aspects, and cognition. Sensory aspects continually resolved and formed the basis for behavior, followed by social interaction and responsiveness, cognition, and speech or communication.

In each of the domains, a few symptoms did not improve in a large percentage of patients. The ability to relate to people requires recognition, understanding of relations (e.g., father and son), emotional bonding, and reciprocity. Only a few patients (29.63%) showed improvement in this owing to a shorter duration of followup. On the three symptoms, namely, lack of fear of danger under the domain of emotional responsiveness, insensitivity to pain within the domain of sensory aspects, and staring into space for long periods of time; 36.84%, 26.32%, and 25% of individuals improved, respectively. We hypothesize that these are interrelated symptoms. For emotional responsiveness, one may require to develop a clear concept of self and the need to protect oneself. This may lead to the identification and perception of a threat which requires higher levels of understanding (cognition) and conditioning for building the association between cause and effect.

Fear of danger is dependent on the perception of pain which may be affected due to sensory processing and integration problems. They may be underresponsive to tactile, vestibular, and proprioceptive inputs. Yet another possibility is their increased attention towards details and intricacies due to which they miss out on the gross information (e.g., they may be so engrossed in looking at a car's wheels or design so that they fail to notice it speeding towards them). This intense observation of minute features or objects could be the underlying cause of what seems to us as staring into space.

Under the speech, language, and communication domain, few individuals improved on the use of nonverbal language (31.58%), decreased use of jargon or meaningless words (27.78%), and decreased pronoun reversals (27.27%). As stated before speech and communication require longer periods of training for individuals with autism. For reducing the use of jargon, the child must understand that what he or she is saying is meaningless and inappropriate. This may not be handled with behavior strategies alone. A higher level of cognition needs to be developed. Pronoun reversals are linked to the deficit in self-identity and the concept of “I”, “Me,” or “You,” which is affected in them. With a longer duration of followup these areas may get addressed.

Within the domain of cognition, unusual memory and savant ability were unchanged as they are intrinsic to the individual with autism and have a complex underlying mechanism of development.

On FIM and Wee-FIM scales there was no statistically significant change, as the FIM scores were maintained after cellular transplantation suggesting preserved independence level for ADL in all the patients after cellular therapy.

### 4.3. Theoretical Basis for the Clinical Improvements Seen after the Intervention

Kevin et al. presented a model of ASD that implicates an early failure to develop the specialized functions of the social brain that is involved in social information processing. They state that due to this early disruption, “an individual with autism must develop in a highly social world without the specialized neural systems that would ordinarily allow him or her to partake in the fabric of social life, which is woven from the thread of opportunity for social reciprocity and the tools of social engagement [30].” We hypothesize that cellular transplantation causes functional restoration of specialized neural systems by neuroprotection, neural circuit reconstruction, neural plasticity, neurogenesis, and immunomodulation. Individual therapies like occupational therapy, psychological intervention, and speech therapy employ the principles of learning to facilitate neural plasticity. In addition, they also provide the opportunity and tools for social engagement. Enhancement of the neural and functional restoration can be optimized by combining these therapies with cellular transplantation.

### 4.4. Adverse Events

There were some minor adverse events, such as headache (3.6%), nausea (10.7%), vomiting (17.9%), pain at the site of injection (7.1%), and pain at the site of aspiration (7.1%) which were reported in few patients immediately after cell transplant procedure. These mild side effects were controlled with medications and resolved within a week. It is important to consider poor communication skills of children with autism while monitoring the symptomatic adverse events.

One (3.1%) patient was marked as minimally worse on CGI-II, due to persistent increase in hyperactivity at three months which did not subside by six months of followup. During this study, we observed that 6 patients (18.7%) showed a transient phase of minimal increase in hyperactivity in the first three months that subsided by six months. In addition this increase in the hyperactivity did not interfere with the overall improvement. The transient increase in hyperactivity may be a result of increased neuronal activity after cell transplantation, changes in daily routine, and exposure to new tasks during therapy sessions. This needs to be further studied in larger sample and controlled studies.

In few patients (9%) there were seizure episodes after cellular transplantation which were controlled with medications. This may be due to activation of epileptogenic focus which already existed in these patients. This is supported by absence of seizures after intervention in the patients with normal EEG befor intervention. It was observed that in patients with no history of seizures but EEG reporting epileptogenic focus, there was increased likelihood of seizures after intervention. It is noteworthy that the seizures did not cause clinical deterioration or hamper beneficial effects of cell therapy.

No other major adverse events were noted over the period of 26 months (mean = 12.7 months).

The overall improvements on ISAA was observed in 29 patients out of 32 (90.6%), and 20 patients out of 32 (62.5%) improved on CGI-I, which was significant. The grading of global improvements on CGI-II showed that 37.5% of patients had very much improvement, 46.8% had much improvement, and 18.8% had minimal improvement.

CGI-III efficacy index provides us with evidence that the intervention may be an effective tool for treatment of autism. 21.9% of patients showed marked therapeutic effect with no side effects, 40.6% patients showed moderate therapeutic effect, and 28% showed minimal therapeutic effect; none of these patients had any major side effects.

### 4.5. PET-CT Scan Evidence

The complex constellation of symptoms of autism cannot be explained by pin pointing to a specific structure or area of the brain. The hypothesis of social brain and theory of mind tasks emphasizes that the symptoms of autism are routed not only in the structural deviations but also in the deficits in neural connectivity [29]. Various attempts have been made to investigate the areas of dysfunction, using various radiological and nuclear imaging techniques [54, 55]. These investigations, however, do not explain the putative neural connectivity deviations causing the symptoms of autism. Functional neuroimaging is thought to give more lucid information about neural connectivity [56]. PET-CT scan and Functional MRI (FMRI) scan are most widely used functional neuroimaging techniques. PET-CT scan of brain is a noninvasive, relatively safe, and feasible modality to record the functional activity of brain. It measures 18-FDG uptake which is related to the glucose metabolism at the cellular level which correlates with the functioning of the area of the brain. PET-CT scan remains a choice of investigation in children with autism due to relative ease of conduction and measurement. PET-CT scan studies in children with autism have earlier identified reduced metabolic activity in bilateral temporal lobes [57, 58]. Another study showed significant hypoperfusion of superior temporal gyrus and superior temporal sulcus in children with autism compared to the control children [33]. These findings are consistent with the theory of social brain. We used PET-CT scan to observe the metabolic activity of the brain before and after cellular therapy. The scan was done in a standardized manner, maintaining similar conditions before scanning to ameliorate confounding factors and therefore the changes in the 18-FDG uptake may be attributed to the intervention.

**Figure 4:**
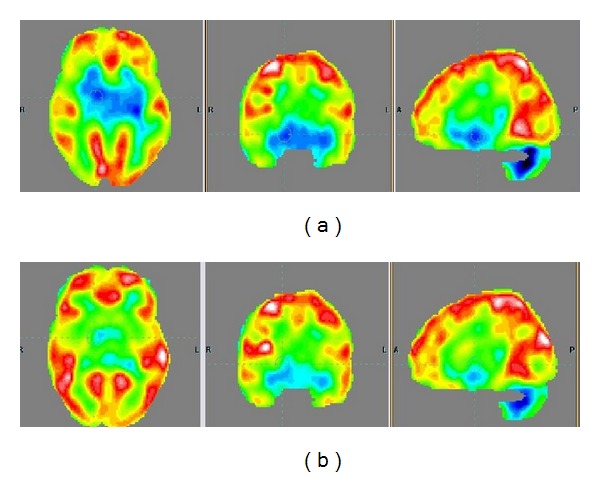
Findings in PET-CT scan before and after cellular therapy. (a) PET-CT scan before intervention showing reduced FDG uptake in the areas of frontal lobe, cerebellum, amygdala, hippocampus, parahippocampus, and mesial temporal lobe. (b) PET-CT scan six months after intervention comparison shows increased FDG uptake in the areas of frontal lobe, cerebellum, amygdala, hippocampus, parahippocampus, and mesial temporal lobe.

A comparative PET-CT scan before and six months after cellular transplantation showed a balancing effect on the metabolism. The areas of hypermetabolism implicated in previous imaging studies [59] showed reduction in metabolism after cellular transplantation, and areas having hypometabolism as suggested by Zilbovicius et al. 2000 [33] showed increased metabolism after cellular transplantation. We hypothesize that immunomodulatory effects and neoangiogenesis causes improved oxygenation and functioning of the damaged neurons. This improves their metabolism which leads to increased FDG uptake in the previously hypofunctional neurons. The paracrine effects and the anti-inflammatory effects also lead to inhibition of hyper functional neurons causing decrease in FDG uptake in the previously hyperfunctional neurons. The exact mechanism still remains unknown. These changes could be clinically correlated with statistically significant reduction of scores of the domains of ISAA scale and reduction in the severity of disease as measured on CGI-I scale (Figure 4).

### 4.6. Limitations and Future Directions

The study is an open label proof of concept. A small sample size, the absence of randomization, and the absence of control group were the limitations. Large scale, multicentre, and randomized controlled trials are recommended. A longer period of followup may be required to further establish the safety and efficacy. Few patients had increased episodes of seizures after the intervention, which were controlled with medications. We recommend preintervention EEG assessments to identify patients with high risk for seizures, after cellular therapy. The requirement and effectiveness of prophylactic antiepileptic medications in such patients must be studied. PET-CT scan was used as evidence in a small number of patients as it was introduced at a later stage, in view of marked clinical improvements. Future studies should consider the use of PET-CT scan as a monitoring tool and substantiate the effects of cellular therapy in autism.

This study presents results of autologous BMMNCs intrathecal administration, still the other routes of administration; types of cells, combination of cells, dosage of cells, and frequency of the transplantation must be explored. It is believed that the effect of stem cells is larger in younger age group due to greater plasticity of the brain and increased availability of precursor cells in the bone marrow. It may be helpful to identify the most responsive age group for cellular therapy.

In this study, one of our concerns was that the improvements observed may be due to various therapies other than cell transplantation. But, several studies have been reviewed for evidence of effects of individual therapies including occupational therapy, sensory integration, behavior therapy, and speech therapy in children with autism. Case-Smith and Arbesman in their review [60] show that these individual therapies have some positive findings, but the evidence is weak due to limitations of small sample size, short followup, lack of evaluation instruments, inadequate measures of treatment fidelity, and inappropriate data analysis. So, according to them the interpretation of the findings of the studies of the individual therapies needs to be with caution. Therefore, the significant clinical improvements seen in our study cannot be attributable to the multidisciplinary therapies alone. Our study demonstrates that cellular therapy has synergistic effect and enhances the effects of multidisciplinary therapies. Hence, we postulate that the combination of cell transplantation with various therapies offers an augmented beneficial response. Future studies may be planned with a control group for these individual therapies.

## 5. Conclusion

Despite accumulating evidence of the safety of cellular therapy, there is a dearth of published human clinical studies. Autism has been in discussion since a long period and the increasing incidence has established a need to find a definitive treatment modality. Therefore, the transition of cellular therapy from benchside to bedside is warranted. Though autologous BMMNCs may not be a cure for autism, they definitely possess the potential to manage overall disease severity and improve the quality of life. This study is a preliminary demonstration of the safety and efficacy of autologous BMMNCs in autism. The minimal invasiveness, simplicity of the procedure, and autologous nature of the cells render it as a promising therapeutic potential. This is the first clinical study on the application of cellular therapy in patients with autism which may provide future directions for larger randomized controlled trials.

## Supplementary Material

Supplementary material consists of scales like ISAA, CGI, FIM and WeeFIM which have been used as outcome measures in our study.

## Figures and Tables

**Table 1:** Demographic data and scores of CGI, ISAA, FIM, and Wee-FIM scales before cell transplantation.

Category	Minimum	Maximum	Average/median	Standard deviation
Demographic data				
Follow-up duration (months)	5	26	12.7	7.1
Age at intervention (years)	3	33	10.49	5.59
Diagnosed since (years)	0	18	7.17	4.20

Baseline scale scores				
CGI-I scale scores	3	6	4.5	0.97
ISAA scale scores	148	160	115.5	24.26
FIM scale scores	48	118	77	18.32
Wee-FIM scale scores	18	110	76	24.06

**Table 2:** Change in the scores of CGI and ISAA before and after intervention.

Scale	Median score before cellular therapy	Median score after the cellular therapy	Test statistics	Statistical significance
CGI-I	4.5	3	*Z* = −3.509	*P* < 0.001*
ISAA scale	115.5	97	*Z* = −4.670	*P* < 0.001*

*Statistically significant (level of significance at *P* < 0.05).

**Table 3:** Change in the ISAA scores of individual domains measured before and after intervention.

ISAA scale domain	Median score before cellular transplantation	Median score after cellular transplantation	Test statistics of Wilcoxon signed rank test for matched pairs	Statistical significance
Social relationship and reciprocity	35.5	13	−4.118	*P* < 0.001*
Emotional responsiveness	23	20	−3.153	*P* = 0.002*
Speech, language, and communication	13	11	−3.989	*P* < 0.001*
Behavior patterns	29	10	−3.126	*P* = 0.002*
Sensory aspects	21	17	−2.409	*P* = 0.016*
Cognitive component	11	8	−3.508	*P* < 0.001*

*Statistically significant (level of significance at *P* < 0.05).

**Table 4:** Adverse events monitored over the entire period of follow-up of 26 months.

Adverse events	Present during the period of follow-up		Absent during the period of follow-up	
	Procedure related	Cellular transplantation related	Procedure related	Cellular transplantation related
Minor	(i) Spinal headache (3.6%) (ii) Nausea (10.7%) (iii) Vomiting (17.9%) (iv) Pain at the site of injection (7.1%) (v) Pain at the site of aspiration (7.1%)	None	(i) Bleeding at the site of injection (ii) Bleeding at the site of aspiration	None

Major	None	(i) Seizures* (9%) (ii) Transient increase in hyperactivity (18.7%) (iii) Persistent increase in hyperactivity till six months (3.1%)	(i) Neurological deficits (ii) Nerve root damage (iii) Parasthesia in lower limb (iv) Loss of sensation in lower limb (v) Loss of motor function in the lower limbs (vi) Hematoma at the site of injection (vii) Hematoma at the site of aspiration (viii) Local infection at the site of injection or aspiration (ix) Meningismus or meningitis (x) Systemic or brain infection (xi) Bowel or bladder incontinence (xii) Respiratory distress (xiii) Cardiac failure	Allergic reaction

*Seizures were considered to be an adverse event when seizures observed were new onset postintervention with no previous history or there was increased frequency or severity of seizures as compared to preintervention.

**Table 5:** Details of three patients who had seizures as an adverse event after cellular therapy.

	Patient I	Patient II	Patient III
Type of seizures (before and after intervention)	Pre-GTC Post-GTC	Pre-No seizure Post-GTC	Pre-No seizures Post-GTC
Number of seizure episodes after intervention	One	Multiple	Two
Duration in months between the cellular therapy and first seizure episode	Six	Four	Three
Duration in months over which seizure episodes recurred	No recurrence	Ten	Three
Medication used for seizure control	Midazolam	Sodium Valproate dose was doubled. Clobazam was discontinued and Lamotrigine was added	Sodium Valproate
Seizure related complications	None	None	None
Effect of seizures on clinical improvement	There was no deterioration in the baseline and the marked clinical improvement was maintained	There was no deterioration in the baseline and the marked clinical improvement was maintained	There was no deterioration in the baseline and the marked clinical improvement was maintained
