# Supplementary material for: Autologous Bone Marrow Mononuclear Cell Therapy for Autism: An Open Label Proof of Concept Study
Source: Stem Cells Int. 2013 Aug 25;2013:623875. doi: 10.1155/2013/623875 (PMC3767048; doi:10.1155/2013/623875)
Supplement: Supplementary file 1 — Supplementary material consists of scales like ISAA, CGI, FIM and WeeFIM which have been used as outcome measures in our study. [file 623875.f1.doc]

Appendix I

*Indian Scale for Assessment of autism*

*Directions:*

Below are given 40 statements which are divided under six domains, please enter “Y” in the appropriate rating for each item of the scale by observing the child and by interviewing the parents in order to assess Autism.

| **Items** | | | | **Rarely** | | **Sometimes** | | **Frequently** | | | **Mostly** | **Always** | |
| --- | --- | --- | --- | --- | --- | --- | --- | --- | --- | --- | --- | --- | --- |
|  |  |  |  | **Upto 20%** | | **21- 40%** | | **41 – 60%** | | | **61 – 80%** | **81 – 100%** | |
|  |  |  |  | **Score 1** | | **Score 2** | | **Score 3** | | | **Score 4** | **Score 5** | |
| ***Social Relationship and Reciprocity*** | | | | | | | | | | | | | |
| *1. Has poor eye contact* | | | |  | |  | |  | | |  |  | |
| *2. Lacks social smile* | | | |  | |  | |  | | |  |  | |
| *3. Remains aloof* | | | |  | |  | |  | | |  |  | |
| *4. Does not reach out to others* | | | |  | |  | |  | | |  |  | |
| *5. Unable to relate to people* | | | |  | |  | |  | | |  |  | |
| *6. Unable to respond to social/environmental cues* | | | |  | |  | |  | | |  |  | |
| *7, Engages in solitary and repetitive play activities* | | | |  | |  | |  | | |  |  | |
| *8. unable to take turns in social interaction* | | | |  | |  | |  | | |  |  | |
| *9. Does not maintain peer relationships* | | | |  | |  | |  | | |  |  | |
| ***Emotional Responsiveness*** | | | | | | | | | | | | | |
| *10. Shows inappropriate emotional response* | | | |  | |  | |  | | |  |  | |
| *11. Shows exaggerated emotions* | | | |  | |  | |  | | |  |  | |
| *12. Engages in self – stimulating emotions* | | | |  | |  | |  | | |  |  | |
| *13. Lacks fear of danger* | | | |  | |  | |  | | |  |  | |
| *14. Excited or agitated for no apparent reason* | | | |  | |  | |  | | |  |  | |
| ***Speech – Language and communication*** | | | | | | | | | | | | | |
| *15. Acquired speech and lost it* | | | |  | |  | |  | | |  |  | |
| *16. Has difficulty in using non-verbal language or gestures to communicate* | | | |  | |  | |  | | |  |  | |
| *17. Engages in stereotyped and repetitive use of language* | | | |  | |  | |  | | |  |  | |
| *18. Engages echolalic speech* | | | |  | |  | |  | | |  |  | |
| *19. Produces infantile squeals / unusual noises* | | | |  | |  | |  | | |  |  | |
| *20. Unable to initiate or sustain conversation with others* | | | |  | |  | |  | | |  |  | |
| *21. Uses jargon or meaningless words* | | | |  | |  | |  | | |  |  | |
| *22. Uses pronoun reversals* | | | |  | |  | |  | | |  |  | |
| *23. Unable to grasp pragmatics of communication ( real meaning)* | | | |  | |  | |  | | |  |  | |
| ***Behavior Patterns*** | | | | | | | | | | | | | |
| *24. Engages in stereotyped and repetitive motor mannerisms* | | | |  | |  | |  | | |  |  | |
| *25. Shows attachment to inanimate objects* | | | |  | |  | |  | | |  |  | |
| *26. Shows hyperactivity / restlessness* | | | |  | |  | |  | | |  |  | |
| *27. Exhibits aggressive behaviour* | | | |  | |  | |  | | |  |  | |
| *28. Throws temper tantrums* | | | |  | |  | |  | | |  |  | |
| *29. Engages in self – injurious behavior* | | | |  | |  | |  | | |  |  | |
| *30. Insists on sameness* | | | |  | |  | |  | | |  |  | |
| ***Sensory Aspects*** | | | | | | | | | | | | | |
| *31. Unusually sensitive to sensory stimuli* | | | |  | |  | |  | | |  |  | |
| *32. Stares into space for long periods of time* | | | |  | |  | |  | | |  |  | |
| *33. Has difficulty in tracking objects* | | | |  | |  | |  | | |  |  | |
| *34. Has unusual vision* | | | |  | |  | |  | | |  |  | |
| *35. Insensitive to pain* | | | |  | |  | |  | | |  |  | |
| *36. Responds to objects / people unusually by smelling, touching or tasting* | | | |  | |  | |  | | |  |  | |
| ***Cognitive Component*** | | | | | | | | | | | | | |
| *37. Inconsistent attention and concentration* | | | |  | |  | |  | | |  |  | |
| *38. Shows delay in responding* | | | |  | |  | |  | | |  |  | |
| *39. Has unusual memory of some kind* | | | |  | |  | |  | | |  |  | |
| *40. Has ‘ savant ‘ ability* | | | |  | |  | |  | | |  |  | |
| ***Result*** | | | | | | | | | | | | | |
| **Total Score** | | | |  | | | | | | | | | |
| **Percentage disability score** | | | |  | | | | | | | | | |
| **How to assess as per ISAA score** | | | | | | | | | | | | | |
| **ISAA score** | | **70** | **71-**  **88** | | **89-**  **105** | | **106-**  **123** | **124 - 140** | | **141-**  **158** | | **>158** |  |
| **Percentage of disability (%)** | | **40** | **50** | | **60** | | **70** | **80** | | **90** | | **100** |  |
|  | | | | | | | | | | | | | |
| ***No Autism*** | ***Mild Autism*** | | | | ***Moderate Autism*** | | | | ***Severe Autism*** | | | | |
| ***<70*** | ***70 to 106*** | | | | ***107 to 153*** | | | | ***> 153*** | | | | |
|  | | | | | | | | | | | | | |

Appendix II

Clinical Global Impression scale

1. **Severity of illness**

Considering your total clinical experience with this particular population, how mentally ill is the patient at this time?

0 = Not assessed 4 = Moderately ill

1 = Normal, not at all ill 5 = Markedly ill

2 = Borderline mentally ill 6 = Severely ill

3 = Mildly ill 7 = Among the most extremely ill patients

1. **Global improvement:**

Rate total improvement whether or not, in your judgement, it is due entirely to drug treatment.

Compared to his condition at admission to the project, how much has he changed?

0 = Not assessed 4 = No change

1 = Very much improved 5 = Minimally worse

2 = Much improved 6 = Much worse

3 = Minimally improved 7 = Very much worse

1. **Efficacy Index:**

Rate this item on the basis of drug effect only.

Select the terms which best describe the degrees of therapeutic effect and side effects and record the number in the box where the two

items intersect.

EXAMPLE: Therapeutic effect is rated as ‘Moderate’ and side effects are judged ‘Do not significantly interfere with patient’s functioning’.

| **Side Effects** | **None** | **Do not significantly interfere with patient’s functioning** | **Significantly interferes with patient’s functioning** | **Outweighs therapeutic effect** |
| --- | --- | --- | --- | --- |
| **Therapeutic effect** |
| **Marked (Vast improvement. Complete or nearly complete remission of all symptoms)** | **1** | **2** | **3** | **4** |
| **Moderate (Decided improvement. Partial remission of symptoms)** | **5** | **6** | **7** | **8** |
| **Minimal (Slight improvement which doesn’t alter status of care of patient)** | **9** | **10** | **11** | **12** |
| **Unchanged or worse** | **13** | **14** | **15** | **16** |

**Not assessed = 00**

Appendix III

Functional Independence Measure – (FIM) Instrument

| Levels | 7 Completely independent (Safely, Timely)  6 Modified independence (Device) | No Helper | |
| --- | --- | --- | --- |
|  | **Modified Independence**  5 Supervision ( Subject = 100%)  4 Minimal assistance ( Subject = 75%)  3 Moderate assistance ( Subject = 50%)  **Complete dependence**  2 Maximal Assistance ( Subject = 25%)  1 Total assistance ( Subject < 25%)  * Do not leave score blank, please score 1 when patient cannot be assessed | Helper | |
|  | **Self Care**   1. Eating 2. Grooming 3. Bathing 4. Dressing ( upper body ) 5. Dressing ( lower body ) 6. Toiletting |  | |
|  | **Sphincter control**   1. Bladder management 2. Bowel Management |  | |
|  | **Transfers**   1. Bed , Chair , Wheel chair 2. Toilet 3. Tub, shower |  | |
|  | **Locomotion**   1. Walk / Wheelchair 2. Stairs |  | |
|  |  |  | W = Walk |
|  |  |  | C = Wheelchair |
|  |  |  | B = Both |
|  |  |  | W = Walk |
|  |  |  | C = Wheelchair |
|  |  |  | B = Both |
| **Motor subtotal score** | | | |
|  | **Communication**  N. Comprehension  O. Expression |  |  |
|  |  |  | A = Auditory |
|  |  |  | V = Visual |
|  |  |  | B = Both |
|  |  |  | V = Vocal |
|  |  |  | N = Non – vocal |
|  |  |  | B = Both |
|  | **Social Cognition**  P. Social interaction  Q. Problem solving  R. Memory |  |  |
|  |  |  |  |
|  |  |  |  |
|  |  |  |  |
| **Cognitive subtotal score** | | | |
| **Complete FIM score** | | | |

Appendix IV

Wee- FIM scale

| **Area** | **Score** | **Age norm** | **Explain reasons for giving this score** | ***Due to injury?** |
| --- | --- | --- | --- | --- |
| **SELF CARE** | | | | |
| 1.Eating |  |  |  | Yes  No |
| 2.Grooming |  |  |  | Yes  No |
| 3.Bathing |  |  |  | Yes  No |
| 4.Dressing– Upper |  |  |  | Yes  No |
| 5.Dressing– Lower |  |  |  | Yes  No |
| 6.Toileting |  |  |  | Yes  No |
| 7.Bladder management |  |  |  | Yes  No |
| 8.Bowel management |  |  |  | Yes  No |
| **Self care total** |  |  |  |  |
| **MOBILITY** | | | | |
| 9.Transfers: Chair/Wheelchair |  |  | Mode: W– Walk C- Wheelchair B- Both | Yes  No |
| 10.Transfers:  Toilet |  |  |  | Yes  No |
| 11.Transfers: Tub/Shower |  |  |  | Yes  No |
| 12.Locomotion:  Walk/ Wheelchair/Crawl |  |  | Mode: W – Walk C- Wheelchair L- Crawl B- Both | Yes  No |
| 13.Locomotion: Stairs |  |  |  | Yes  No |
| **Mobility total** |  |  |  |  |
| **COMMUNICATION** | | | | |
| 14.Comprehension |  |  | Mode: A – Auditory V - Visual C - Both | Yes  No |
| 15.Expression |  |  | Mode: V – Vocal N - Non-vocal B - Both | Yes  No |
| **SOCIAL COGNITION** | | | | |
| 16.Social interaction |  |  |  | Yes  No |
| 17.Problem solving |  |  |  | Yes  No |
| 18.Memory |  |  |  | Yes  No |
| **Cognition total** |  |  |  |  |
| **WeeFIM® total** |  |  |  |  |

**WeeFIM® LEVELS**

***NO HELPER***

**7** Complete Independence (Timely, Safely)

**6** Modified Independence (Device)

***HELPER – Modified Dependence***

**5** Supervision

**4** Minimal assistance (subject = 75% or more)

**3** Moderate assistance (subject = 50% or more)

***Helper – Complete Dependence***

**2** Maximal assistance (subject = 25% - 49%)

**1** Total assistance (subject = 0% - 24%)
